# The nanomorphology of cell surfaces of adhered osteoblasts

**DOI:** 10.3762/bjnano.12.20

**Published:** 2021-03-12

**Authors:** Christian Voelkner, Mirco Wendt, Regina Lange, Max Ulbrich, Martina Gruening, Susanne Staehlke, Barbara Nebe, Ingo Barke, Sylvia Speller

**Affiliations:** 1 Department Science and Technology of Life, Light and Matter, University of Rostock, Albert-Einstein-Str. 25, 18059 Rostock, Germany; 2 Institute of Physics, University of Rostock, Albert-Einstein-Str. 23, 18059 Rostock, Germany; 3 Department of Cell Biology, Rostock University Medical Center, Schillingallee 69, 18057 Rostock, Germany

**Keywords:** cell adhesion, membrane fluctuations, osteoblast, plasma membrane nanomorphology, scanning ion conductance microscopy (SICM)

## Abstract

The functionality of living cells is inherently linked to subunits with dimensions ranging from several micrometers down to the nanometer scale. The cell surface plays a particularly important role. Electric signaling, including information processing, takes place at the membrane, as well as adhesion and contact. For osteoblasts, adhesion and spreading are crucial processes with regard to bone implants. Here we present a comprehensive characterization of the 3D nanomorphology of living, as well as fixed, osteoblastic cells using scanning ion conductance microscopy (SICM), which is a nanoprobing method that largely avoids mechanical perturbations. Dynamic ruffles are observed, manifesting themselves in characteristic membrane protrusions. They contribute to the overall surface corrugation, which we systematically study by introducing the relative 3D excess area as a function of the projected adhesion area. A clear anticorrelation between the two parameters is found upon analysis of ca. 40 different cells on glass and on amine-covered surfaces. At the rim of lamellipodia, characteristic edge heights between 100 and 300 nm are observed. Power spectral densities of membrane fluctuations show frequency-dependent decay exponents with absolute values greater than 2 on living osteoblasts. We discuss the capability of apical membrane features and fluctuation dynamics in aiding the assessment of adhesion and migration properties on a single-cell basis.

## Introduction

Osteoblasts are bone-mineralizing cells situated inside the matrix boundaries of the osteoid. They can release matrix vesicles containing calcium and phosphate, eventually leading to precipitation and growth of bone mineral [1–2]. They adhere to and spread on a wide spectrum of pristine and coated material surfaces, such as titanium and polyallylamine [3–5], gelatin–nanogold [6], polyelectrolytes and arginylglycylaspartic acid peptides [7], or extracellular matrix proteins [5,8–9]. Especially on the nonphysiological surfaces of permanent implants, the settling of cells and the swift formation of large adhesion interface areas are desired. Regarding this, a variety of surface coatings was assessed. Relevant parameters may be the zeta potential and pre-adsorbed cell adhesion proteins from the serum of the medium [4,10]. Before osteoblasts start the adhesion and spreading programs, they settle on the surface of the material. Apart from cell biologic parameters, the adhesion interface area and the speed of its formation provide insights concerning the biocompatibility of the surface with regard to osteoblastic cells [11].

Dynamic remodeling of the cytoskeleton is the basis for shape adaptation and migration for many mammalian cell types [12–13]. Migrating and spreading cells form flat, actin-supported, organelle-free regions, referred to as lamellipodia, and other features that may expand their attachment area [14]. A physical coupling of adhesion molecules to the actin polymerization machinery has been determined [15]. In the course of adhesion-related cell processes considerable nanoscale rearrangements take place inside and on the surface of the cells. Some of them are difficult to address by optical imaging methods due to limited resolution or unduly high light exposure. Scanning probe microscopy is an option to study the membrane surface nanoscopically without dye labeling or laser light exposure.

In scanning probe microscopy a nanoprobe is kept at a constant distance from the sample surface by maintaining a local interaction signal constant via a feedback loop [16]. If the interaction signal is a force, pressure is applied to the sample. This is the case when using atomic force microscopy (AFM), giving rise to substantially depressed apparent heights on living and fixed cells [17]. Typically, mammalian cells exhibit Young's moduli in the range of 1 to 10 kPa while AFM probe pressures may correspond to ca. 10 MPa, assuming 1 nN loading force and 5 nm tip radius. Though the resulting artificial depression depths on the cellular membranes are about half a micrometer already at 1 nN, AFM is useful to measure the cortical actin network underneath the membrane [18].

Regarding the investigation of the membrane itself, there are intricate issues concerning the applied forces during cell surface–nanoprobe interactions. For example, cellular responses, such as cytoskeleton rearrangements and repair of the extracellular matrix, may be triggered. Thus, the native state of the membrane may remain obscure. A localized ion current flowing through a nanopipette probe represents a suitable non-invasive interaction, which is exploited in scanning ion conductance microscopy (SICM) [19–21]. SICM is well suited to probe soft and responsive surfaces, such as those of living cells. The applied pressure is only a few hundred pascals and results from the hydrostatic pressure of the fill level of the nanopipette [22]. The ion current drops during the probe–sample approach, because the effective area for the ion trajectories becomes smaller. This effect is referred to as current squeezing. SICM is the only method capable of nanoscopic three-dimensional imaging of living cells without the application of dye labels or other modifications.

Though SICM was developed already in 1989 [19], it was not much exploited until the method was used to image a number of murine and human cell lines [23]. Meanwhile, nanomorphologies of living cells have been recorded on a number of mammalian cell types, such as cardiomyocytes, fibroblastic cells, neurons, as well as renal and epithelial cells [24–27]. Osteoblast surfaces have not been addressed, neither on fixed nor on live cells. We investigate the nanomorphologies of osteoblast-like cells (MG-63) adhered on glass and amine-functionalized surfaces in live and fixed states. Note that by “nanomorphology” we refer to structures laterally not smaller than ca. 50 nm, since the lateral resolution is limited by the opening of the nanopipette. Our studies address the initial phase of adhesion well before the release of matrix vesicles in the collagen matrix. Our results include characteristic sheet-like protrusions, so-called ruffles. Their appearance on the osteoblast cell rims mostly vanishes when a large adhesion area is established, resulting in a smooth apical plasma membrane surface. Several other morphological and dynamic parameters are evaluated, for example, cell edge heights, membrane surface roughness, and membrane fluctuations, and discussed with respect to cellular functions.

## Results and Discussion

**Figure 1:**
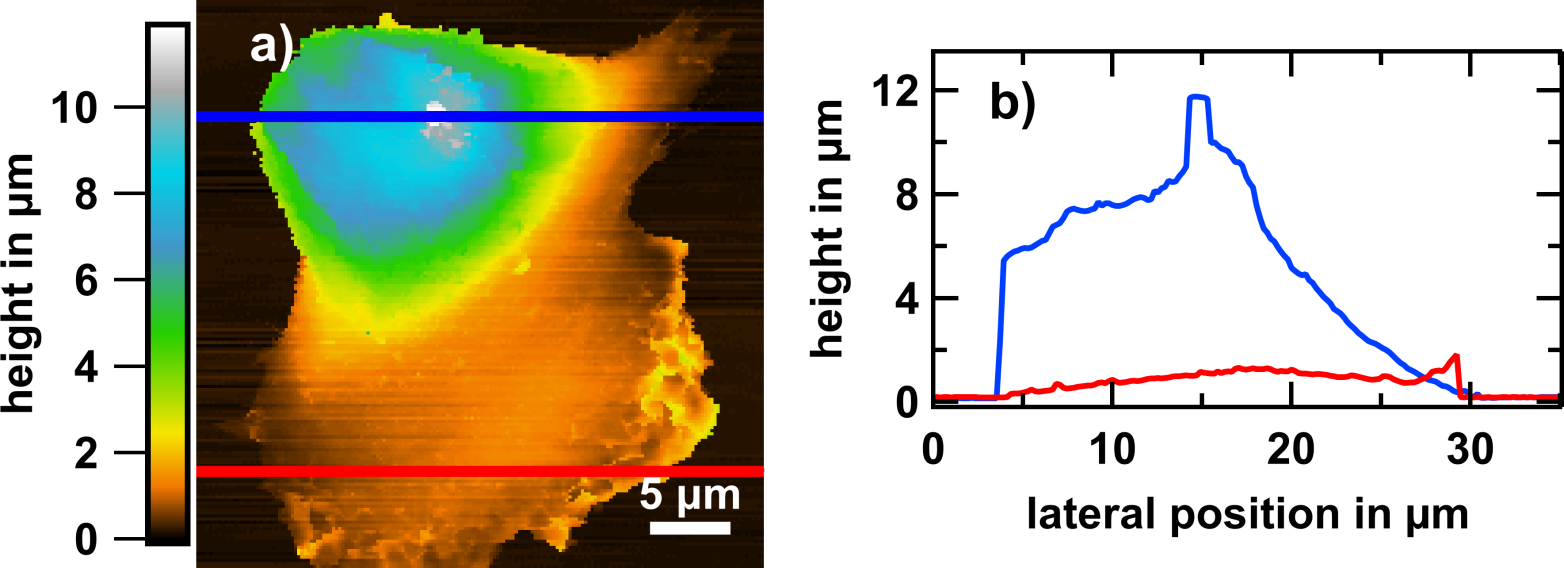
SICM analysis of a fixed MG-63 osteoblastic cell. (a) Overview morphology of the cell on glass after 24 h of culturing. The polar morphology shows that the cell was migrating towards the bottom of the image. (b) Exemplary height profiles of the trailing edge (blue) and the leading edge (red) along the horizontal lines indicated in (a).

In Figure 1 we show a typical overview SICM image of a whole osteoblast in the fixed state. The morphology is polar, that is, a flat region (lamellipodium) at the “leading” side and a bulkier region containing the nucleus at the trailing side were formed. The height of the bulky side of the shown cell is 8 µm while that of the lamellipodium is 500 nm. However, the dimensions and the extent of polarity vary among different cells (not shown). With time, the adhesion interface increases and polarity gets less distinct. Furthermore, the cell exhibits a pronounced elevation of apparently 2 µm with a width of 1 µm (see blue graph in Figure 1b). Such a SICM signature is compatible with that of a primary cilium [28].

### Dorsal ruffles

**Figure 2:**
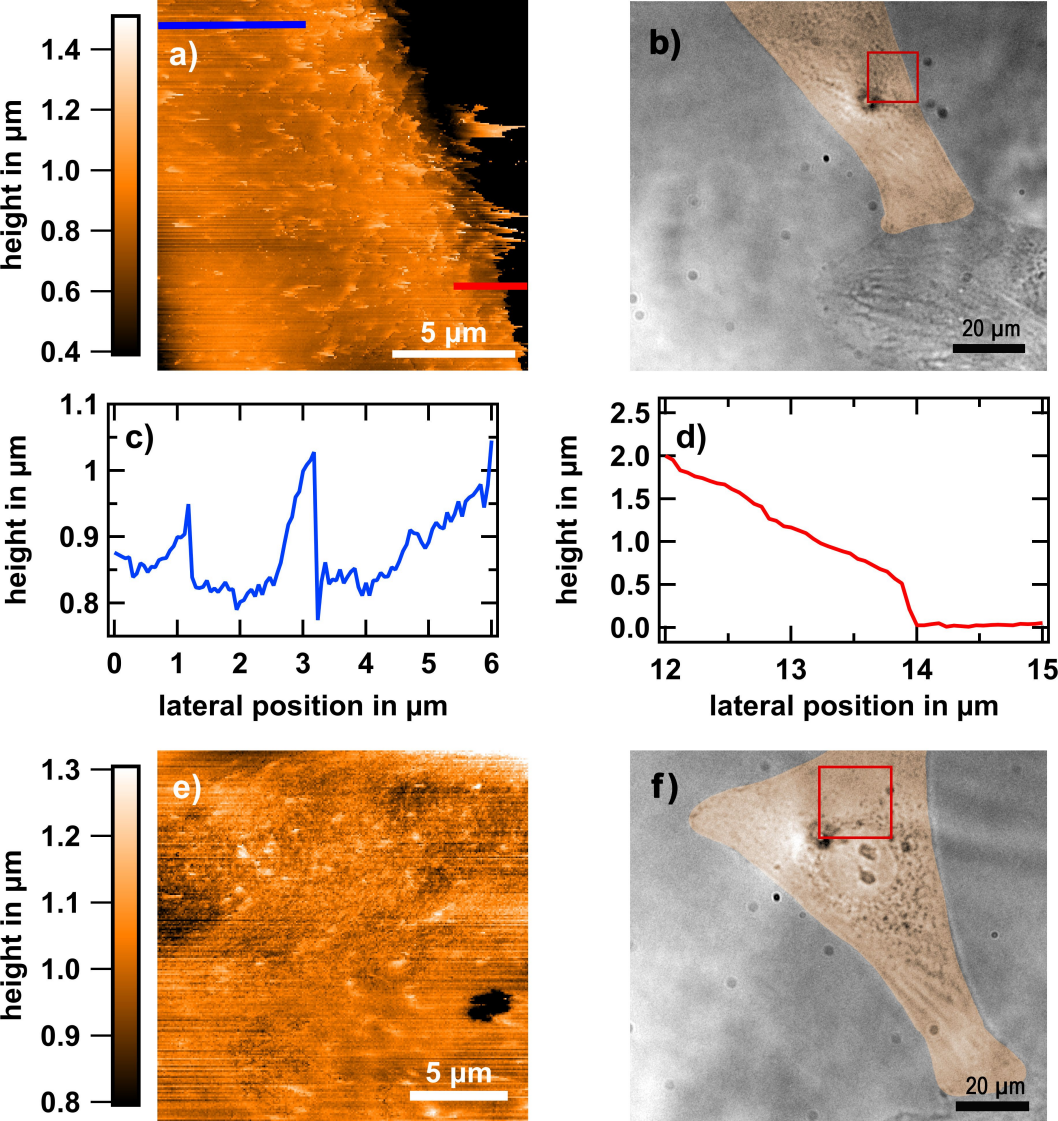
Living osteoblast cell adhered to glass after 24 h of culturing. (a, e) SICM topography images. The acquisition time was 68 min per image. Cell membrane protrusions, so-called ruffles, can be seen as bright spots. (b, f) Corresponding bright-field microscopy images of the living cell taken prior to the SICM measurements. The cell has been digitally stained in orange for better visibility. The areas marked in red were measured with the SICM. (a) Sheet-like protrusions (ruffles) that mostly orient to the cell edge (right side in the image) are visible. The streaky appearance corresponds to the fast scan direction and the orientation of the ruffles is stable upon changing from forward to backward scanning. (c) Height profile along the blue line indicated in (a). Typical feature heights are several hundred nanometers. (d) Line profile at the cell rim, as indicated in (a). A sudden jump from the cell surface to the flat glass surface is visible, which we refer to as the step edge height (here roughly 500 nm).

The morphologies of osteoblast membranes reveal sheet-like protrusions, so-called ruffles. In general, they cover the whole cell surface. They are quite thin (similar to the thickness of filopodia of ca. 100 nm) and flexible. Figure 2a shows an example of a typical SICM topography of the border region in the live state (see Figure 2b for the corresponding bright-field microscopy image). Features with lateral dimensions of approximately 1 µm × 0.8 µm protruding 100–300 nm from the surrounding (Figure 2c), at a density of 0.3–0.5 features per μm^2^, are observed. The characteristic dynamics of the sheets is fast compared to the acquisition leading to temporal undersampling. This results in distortions, which is particularly evident in Figure 2e where the ruffles appear as blurred bright spots.

**Figure 3:**
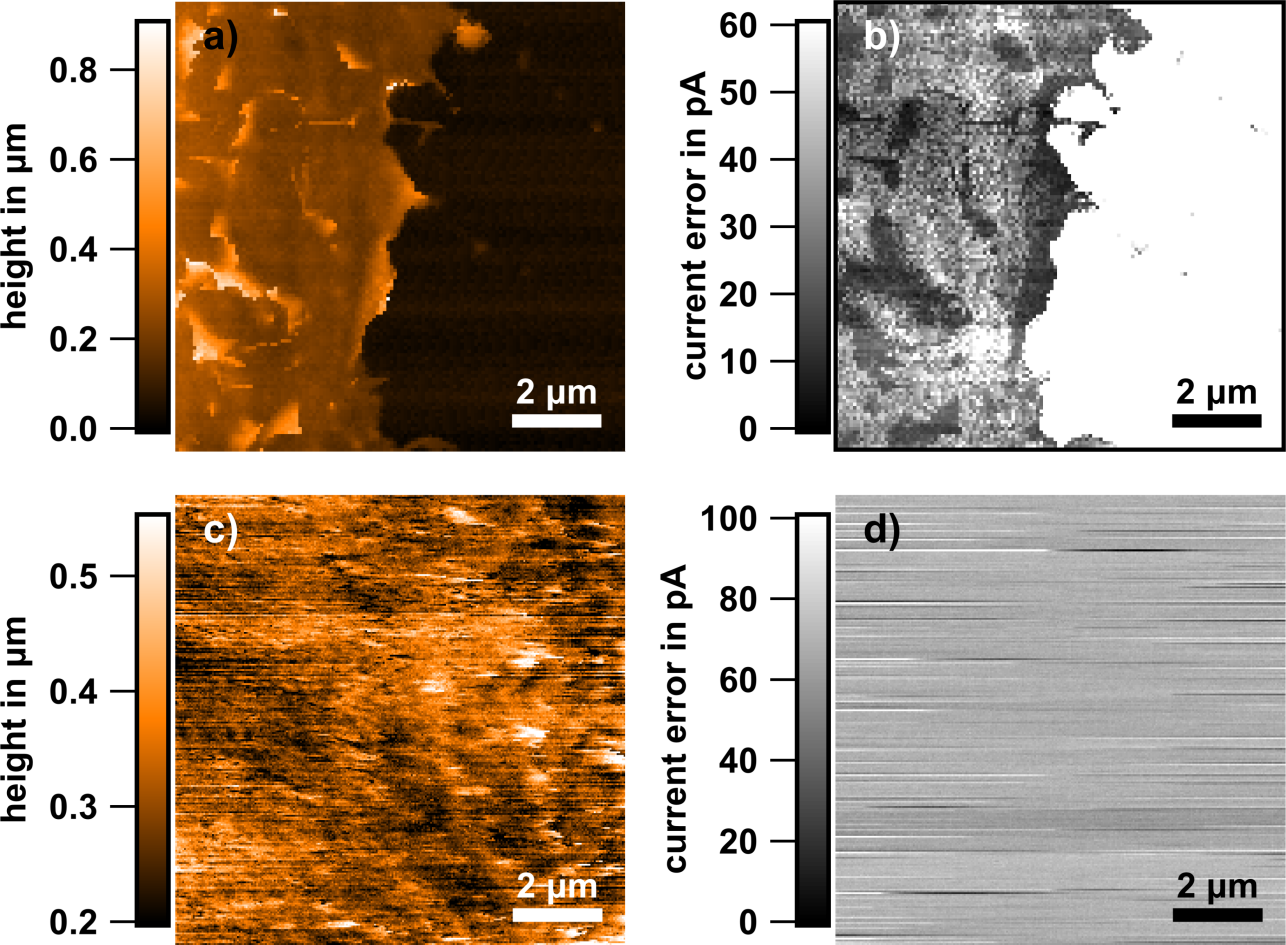
Comparison of living and fixed MG-63 cells by SICM. (a) Osteoblast adhered to glass after fixation (3 h). SICM topography image of the edge of the cell. The ruffles are clearly visible. (b) The corresponding current error image. Note that the ruffles appear to lead to smaller error values, resulting in a contrast in the error map. (c) SICM topography of a living osteoblast adhered to glass (24 h). (d) The corresponding current error map shows that there is no error contrast in the live state.

This becomes obvious when the live-cell dynamics is suppressed upon fixation of the osteoblasts with 4% paraformaldehyde (PFA). Figure 3a shows an example of a respective SICM topography. Now, the ruffles exhibit a clearer shape and resemble similar features to those observed with electron microscopy [29–30]. Our data reveal that the ruffles are ragged. They resemble fins and they confine an acute angle towards the membrane. Frequently, they show twisting. In the fixed state the angle is not very variable and the ruffles do not exhibit flapping when the fast scan direction is reversed.

Similar sheet-like structures have been observed for dendritic cells. It has been shown that these structures serve as catchers for T cells [31]. Ruffles have been described on osteoblasts upon exposure to parathyroid extract [32] and after internalization of polymer or metal particles [33], on fibroblastoid cells [29,34], on breast cancer cells [30], and on keratinocytes [35]. In general, these features are associated with migration, receptor internalization, and micropinocytosis [30,36–37]. Membrane ruffling is regulated by a distinct signaling pathway [38] and the supporting actin is denser and more cross-linked [35] compared to flat membrane regions. Three types of ruffles can be discriminated, namely (linear) dorsal, peripheral, and circular dorsal ruffles [36]. Colocalization with hyaluronan synthase has been found in a breast cancer cell line (MCF-7) [30].

Usually the error current, that is, the deviation between setpoint and actual current at the time the approach is stopped is higher on hard materials than on soft materials (see, e.g., the white area on the right side of Figure 3b, corresponding to glass). The reason is the different slope of the current–distance curves [38]. Because of this, the delay between current sensing and motion termination results in a larger error signal on hard materials than on soft materials. Thus, the comparison between ion current error images of living and fixed cells can provide insight into the protein content in the ruffle volume, because fixation leads to crosslinking of proteins resulting in a higher Young's modulus.

The fixed ruffles exhibit an extremely low ion current error (Figure 3b), lower than that of the surrounding membrane. PFA usually increases the Young's modulus of cell surfaces, for instance, from 3.5 to 18 kPa in fibroblasts [39]. Therefore, the lower ion current error appears counterintuitive regarding the material properties. However, considering the fin or springboard morphology of ruffles, the elasticity may not result from changes of material properties only but also from the flexible shape. Thus, the extremely low error probably points towards lower stiffness of the ruffle structure, even when a harder (fixed) protein content is present. Note that for living cells such contrast is absent as can be seen in Figure 3c and Figure 3d.

**Figure 4:**
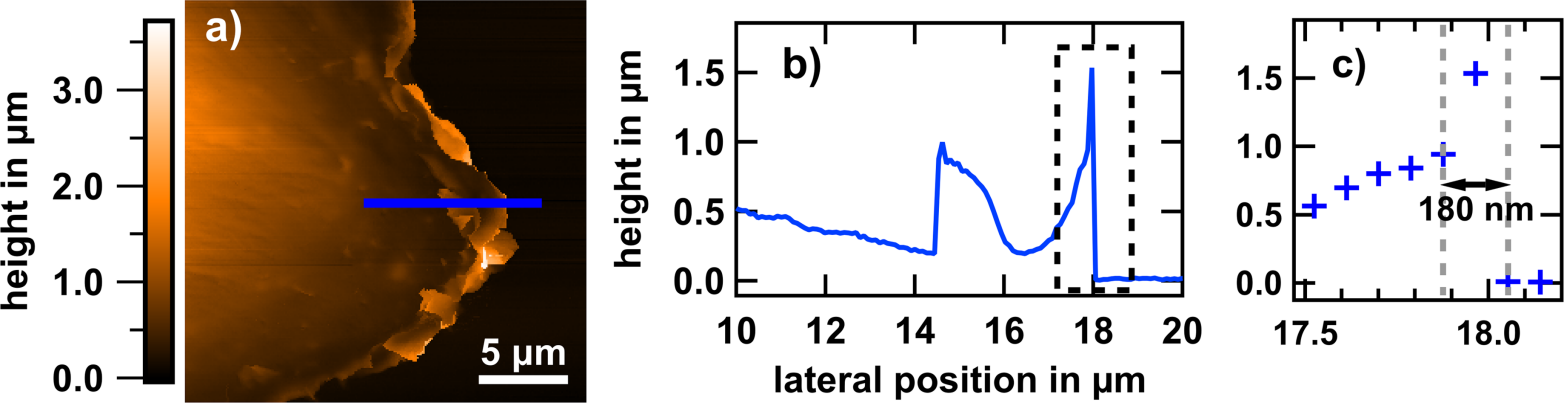
SICM analysis of a fixed cell adhered for 24 h to a 10 nm Au layer. (a) SICM topography of the peripheral ruffles of the cell. Such ruffles can exhibit extensions of more than 1 μm as shown by the blue line in (a), the height profile of which is shown in (b). (c) Expansion of the section highlighted with the dashed lines in (b). Note that the upper thickness limit of this peripheral ruffle is 180 nm, assuming that it forms an angle of 90° with respect to the glass surface.

Figure 4a shows an example of a peripheral ruffle. Peripheral ruffles sometimes are attributed to loosened lamellipodia. Such loosening and the subsequent retraction of lamellipodia towards the cell body has been shown for keratinocytes [35]. Either the attachment of the lamellipodium via integrin was not successful or such processes may serve the cell to attain flexibility of structures and thereby responsiveness. Dynamic remodeling of structures, which requires a continuous balance between assembly and disassembly, is well known for cytoskeletal fibers.

**Figure 5:**
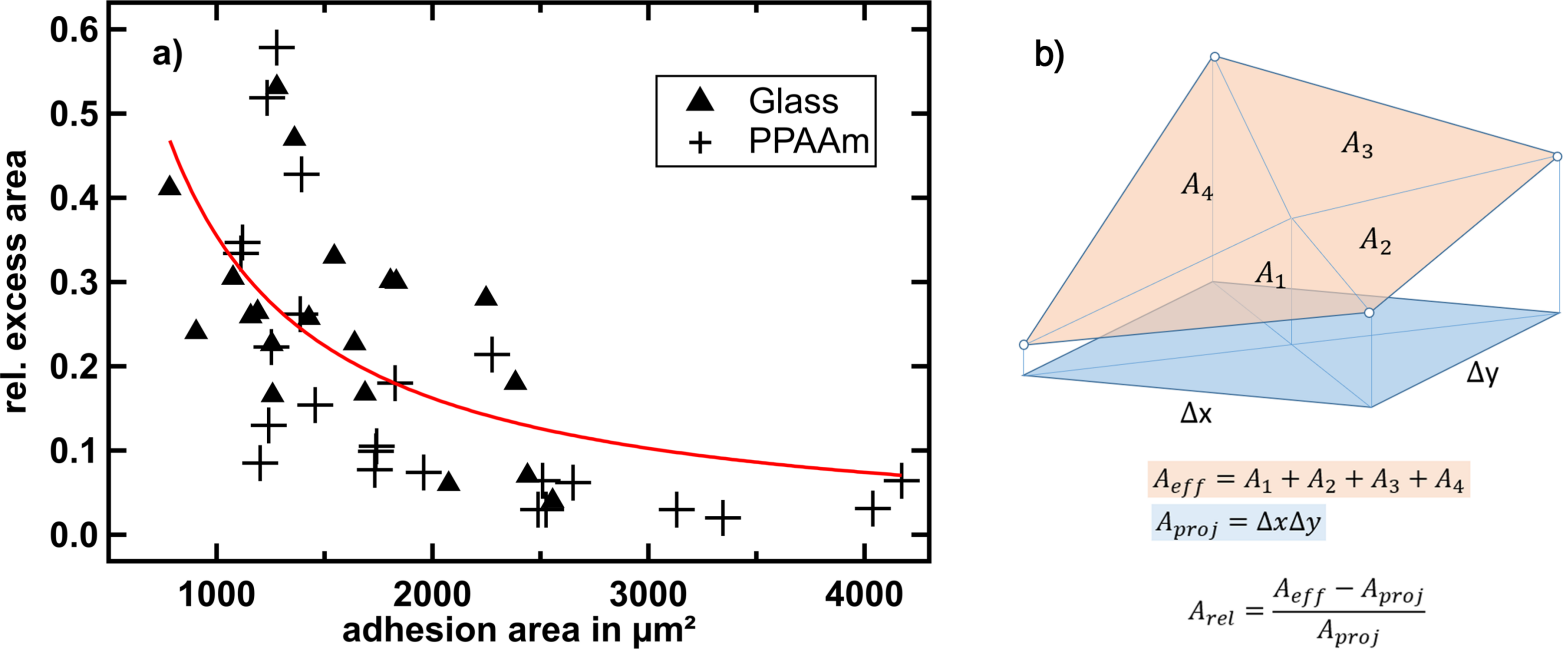
(a) Correlation plot of the relative excess surface (ordinate) and the total adhesion interface area (abscissa) 3 h after seeding. A total of four different cell passages were investigated. Triangles mark values for cells on glass and crosses mark values for cells on the PPAAm coating. On each cell, three edge locations were chosen randomly. The values depicted in the graph are the arithmetic mean of the corresponding relative excess areas. An anticorrelation becomes evident (red graph). (b) Illustration of the effective surface area (orange) and the corresponding projected surface area (blue). The white dots represent height data points.

To quantify the excess membrane of the cells, we fixed the cells already 3 h after adhesion. The reason is that most of the spreading happens in the early phase after initial adhesion [40]. In order to quantify the excess membrane associated with dorsal ruffles, we focused on local frames in the vicinity of the cell rim, not on the large-scale cell morphology. We define the excess surface *A*_exc_ as the difference between the effective and the projected surface area (*A*_eff_ – *A*_proj_). The effective surface is the undulated surface area of the three-dimensional function *z*(*x*,*y*) as determined from SICM topography images,


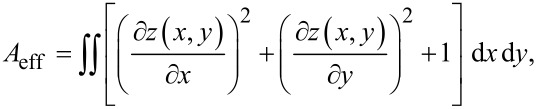


and the projected area is the frame or base area *A*_proj_ = ∫∫d*x*d*y* (Figure 5b). The relative excess surface is then


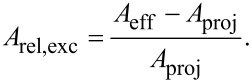


Figure 5a shows the relative excess surface as a function of the adhesion area of the whole cell, *A*_rel,exc_(*A*_adh_). The total adhesion area *A*_adh_ was determined via bright-field microscopy images. Relative excess surfaces between 5% and 60% were found assessing ca. 40 cells. Although the data is substantially scattered, we can observe a clear anticorrelation, namely 
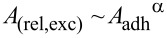
 with α = −1.13 ± 0.25, obtained by least square fit and indicated by the red curve in Figure 5a. This means that a larger adhesion interface area comes along with a smaller excess surface (i.e., a lower number of ruffles or less extended ruffles).

This could mean that excess membrane was “consumed” in more anisotropic or spread cell shapes to compensate for the increased surface-to-volume ratio in the course of spreading. Ruffles could serve as a membrane reserve prior to formation of large adhesion interfaces. This would be compatible with the observation that there are less ruffles found on lamellipodia or rim regions than in more central regions on the cell. For keratinocytes an anticorrelation between ruffling and lamellipodia persistence has been reported [35], supporting this scenario. Another option is that ruffles and excess membrane are signs of former unsuccessful spreading or deliberate lamellipodia loosening, for instance, for the purpose of resumption of migration [15].

Having a look at the substrate-specific data, cells with extremely large adhesion interface areas (more than 2600 µm^2^) are only found on plasma-polymerized allylamine (PPAAm) layers. Nevertheless, the span of measured adhesion areas is large. This may indicate that either the PPAAm layers on our glass surfaces are heterogeneous or that cells do not always respond to it. Another origin of the large spreading may be that, depending on the stage in the cell cycle, the adhesion program may get more or less priority. It has been shown that isolated cells do not synchronize their cell cycle in contrast to cells in groups [41] or in tissues [42–43]. Hence, each cell may be in a different stage. This may contribute to different spreading speeds as observed on PPAAm. According to Pu et al. [44], MG-63 osteoblast-like cells are to 62%, 18.6%, and 19.4% in the G1, S, and G2 phases, respectively. On average (analyzing 44 cells and taking three samplings on each cell) the excess surface on glass (0.25 ± 0.03) is larger than on PPAAm (0.17 ± 0.04). A slightly positive zeta potential, such as measured for PPAAm on titanium, has been figured out to increase the spreading speed [4,45]; our tentative observation of larger maximal adhesion areas on PPAAm than on glass substrates is compatible with these earlier findings. Our results are not compatible with [46], where ruffles have been predominantly observed on primary rat osteoblasts cultured on bioglass, which is less negative in zeta potential compared to quartz glass.

### Membrane holes and smaller protrusions

**Figure 6:**
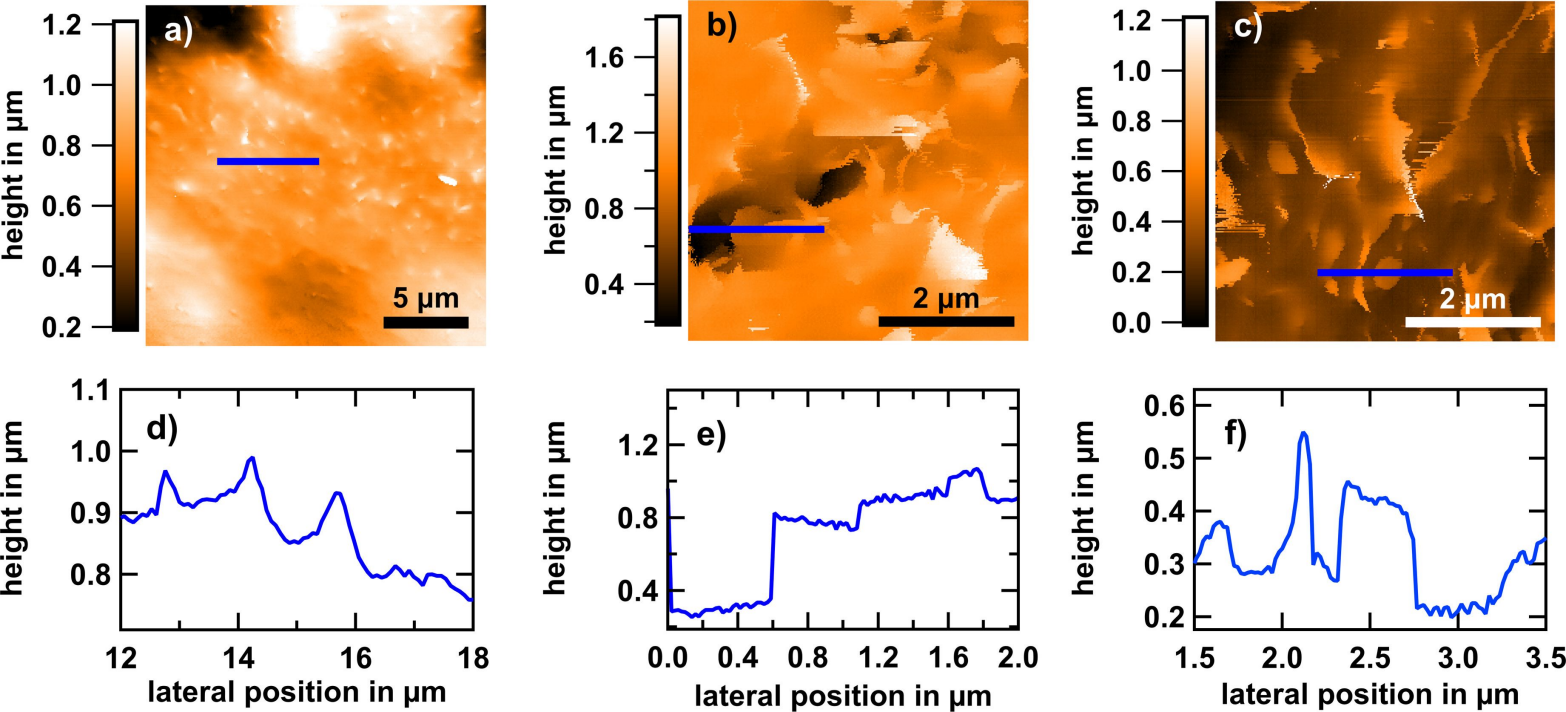
Other membrane features observed by SICM on fixed osteoblastic cells on glass (24 h). (a) Circular tail-like protrusions in the vicinity of the cell nucleus. These tail-like features are characterized by heights of less than 100 nm and seem to show no flapping, which results in almost orthogonal orientation with respect to the cell membrane. (b) Depressions or holes in the cell membrane of ca. 500 nm depth. Similar features in literature are attributed to macropinocytosis events [47]. They may result from the collapse of circular dorsal ruffles. (c) Pancake-like structures, which were rarely observed, show plateau-like characteristics. The heights observed are similar to those of dorsal ruffles. (d–f) The corresponding line profiles.

Though the ruffles are the most prominent feature we observe on the osteoblastic cells, further membrane features, such as holes and circular protrusions, have been encountered. Circular tail-like protrusions were sometimes observed along with the ruffles (Figure 6a). They measure below 100 nm in length. It is difficult to conclude whether those are microvilli or filopodia, or just an early form of ruffles. Microvilli, among others, serve to enhance the exchange of substances with the extracellular medium (through absorption and secretion) while filopodia are used to explore the environment of an adhering cell on a surface, especially if it exhibits some roughness or edges.

Occasionally, we observed depression or hole features in the membrane. They are between a few hundreds of nanometers and one micrometer deep and mostly occur in close vicinity of extensive ruffles (Figure 2e and Figure 6b). Since these holes show lateral dimensions of several hundred nanometers, we can expect the measured depths to be close to the actual value. This is due to the fact that the used nanopipettes have a very high aspect ratio (the cone half angle is ca. 4°) with a small opening diameter (below 80 nm). The features may be related to (macro)pinocytosis, that is, the cells take up smaller or bigger volumes of the extracellular medium. Indeed, ruffling, especially its circular form, has been associated to pinocytosis [30,36–37]. Linear ruffles may be regarded as precursory structures to circular ruffles, and the latter perform pinocytosis [36–37]. The lower incidence and the different dimensions of the circular ruffles (observed as holes with a more or less pronounced “turtleneck”) is compatible with the assumption that they are much more transient, that is, they occur transiently before the extracellular medium is taken up and close shortly after. In SICM observations we found three times more (on ca. 50 cells) structures resembling circular dorsal ruffles, which is consistent with our SEM observations, where we found only one circular dorsal ruffle among 20 cells (not shown).

Rarely, pancake-shaped structures are seen on the membrane, such as those shown in Figure 6c. Since they also measure ca. 200 nm in height, these elevations are definitely no lipid rafts. They could be ruffles that are not anchored sideways to the membrane but somewhere more central underneath; note that the nanopipette of the SICM has no access to hollows underneath other surfaces.

**Figure 7:**
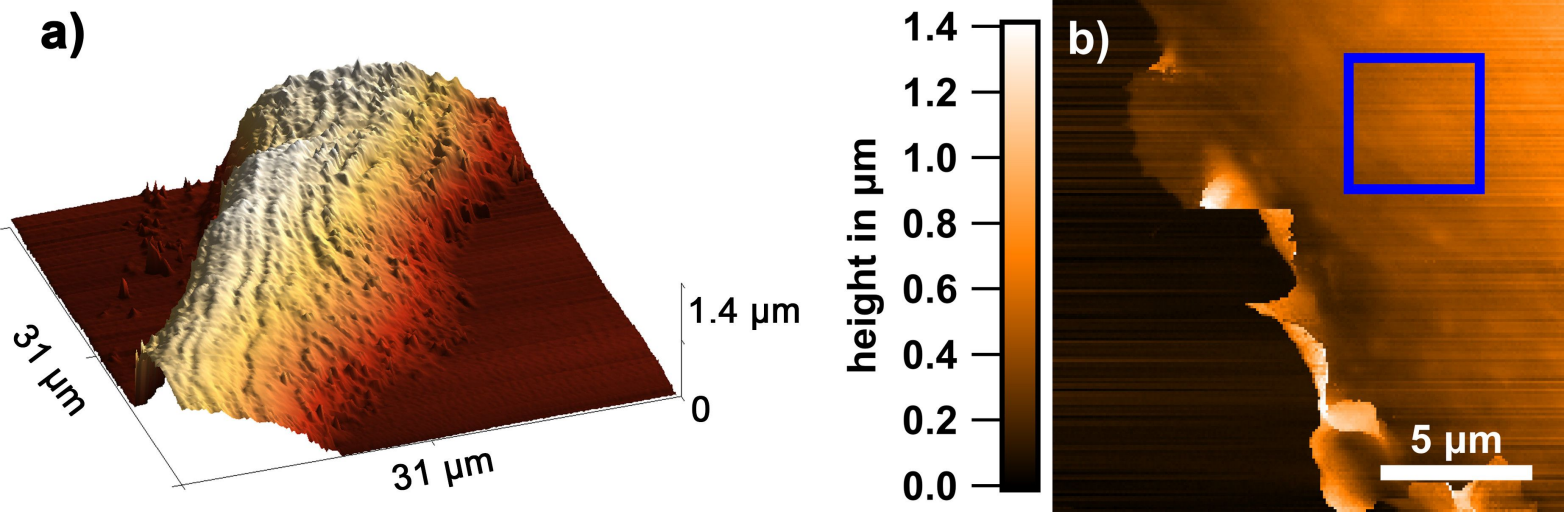
Further membrane features and apparently featureless regions observed by SICM. (a) Pseudo-3D view of a living osteoblastic cell on glass after ca. 5 h. Longitudinal wrinkles that follow the macroscopic shape of the cell are developed. They protrude 100–500 nm from the cell surface. (b) Topography image of a largely featureless membrane region at the edge of a fixed cell. The area marked in blue was exemplarily chosen to determine the 2D-rms roughness value of about 17 nm. Note that a plane was subtracted from the dataset in advance to eliminate the tilt.

Longitudinal shallow wrinkles with periodicities of around 500 nm occur on elongated cells or on larger extensions of them (Figure 7a). Since they come along with anisotropic shapes and the wrinkles are usually aligned parallel to the long side of the cell or its extension, we suspect that they are related to the cytoskeleton underneath, that is, the membrane may cling to the proximate fiber network.

Largely featureless regions, free of ruffles and other membrane structures, are scarce; they show waviness on the mesoscopic scale and a 2D-rms roughness value of 17 nm on the nanoscopic scale, as illustrated in Figure 7b. We find it noteworthy that hardly any filopodia were formed at cellular rims, while this type of extensions was frequently observed on rough or microstructured surfaces.

### Ruffles observed by SEM

**Figure 8:**
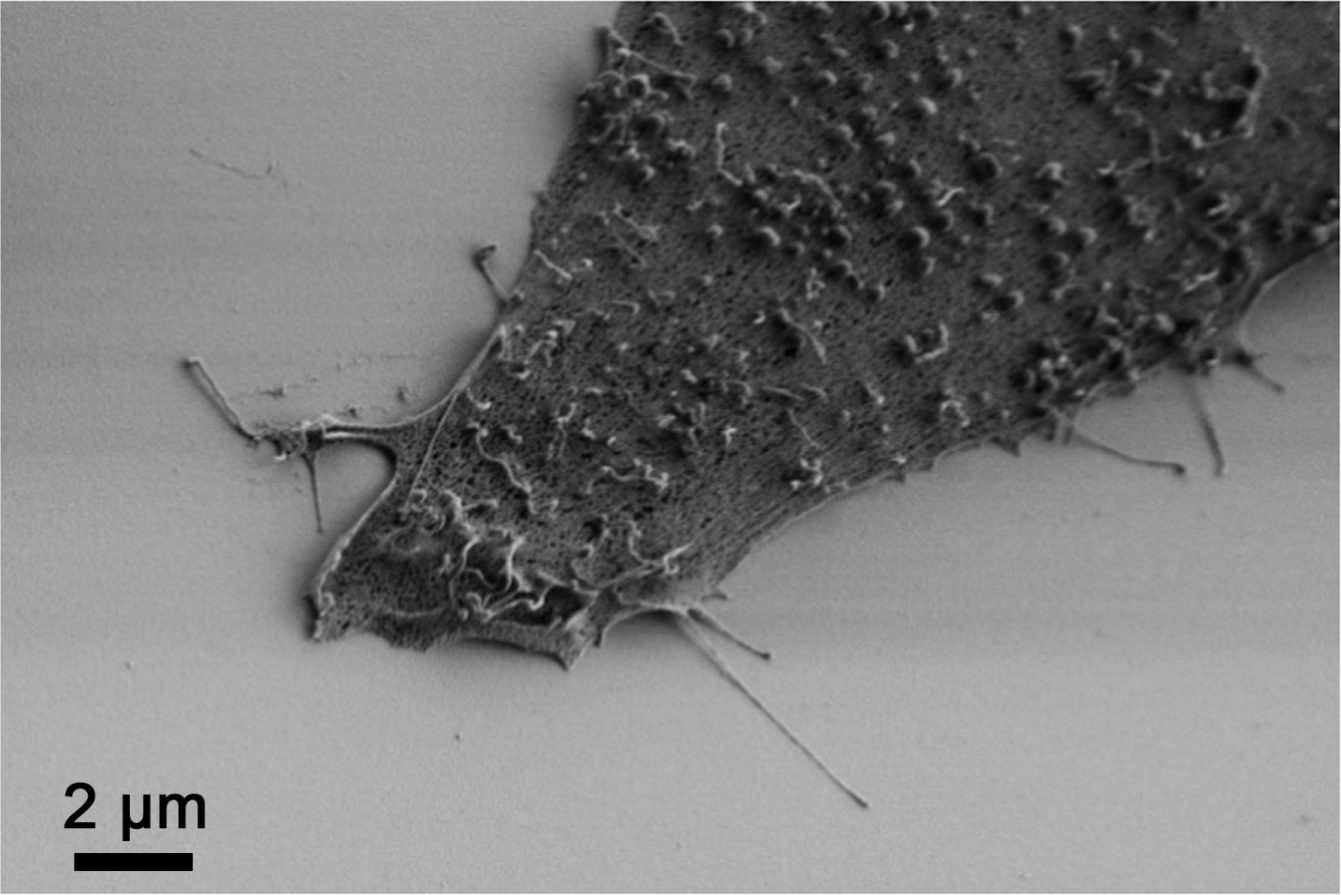
Scanning electron microscopy of an MG-63 osteoblast after 3 h of adhesion to titanium. The ruffles on the cell membrane are visible. (FE-SEM SUPRA25, 1 kV, 30° angle, 100 nm Ti on Si wafer, fixation by 2.5% glutardialdehyde (GA), acetone series, critical point drying).

Osteoblastic cells have been studied extensively by scanning electron microscopy (SEM) [32–33,46]. Electron microscopy can yield nanoscopic resolution but requires invasive preparation such as critical point drying and deposition of thin Au layers. This enables the observation of cell surfaces containing membrane protrusion features; however, apart from [32], no clear ruffles as observed by SICM are recognizable. When no Au coating was applied on the cells of the same osteoblastic cell line (MG-63) that were fixed after 3 h of adhesion on titanium and afterwards critical point dried, the ruffles remain clearer to see (Figure 8).

There are two issues regarding the contrast discrepancy between SEM and SICM that cannot be fully excluded to play a role. By means of critical point drying, volume changes of the cell throughout the preparation procedure for SEM are believed to be largely avoided. This might not fully hold at the nanoscale and some membrane protrusion features could get altered during cell death and the drying procedure. Thus, an option to explain the difference between SEM and SICM images is that the ruffles might be too fragile and are retracted or collapse upon critical point drying. However, especially attractive van der Waals forces might lead to the attachment of ruffles on the membrane surface and, hence, their deformation under vacuum conditions. In contrast to that, the attractive surface forces are screened in physiological medium by ions, leading to undistorted ruffles. Indeed, plenty of worm-like structures are typically observed on osteoblastic cells in SEM images, which could be bent microvilli or residual ruffles.

In summary, plasma membranes in the rim region tend to appear slightly smoother (less ruffled) on cells that have formed large adhesion interface areas. This may indicate that membrane supply is an issue during faster spreading.

### Rim edge heights

**Figure 9:**
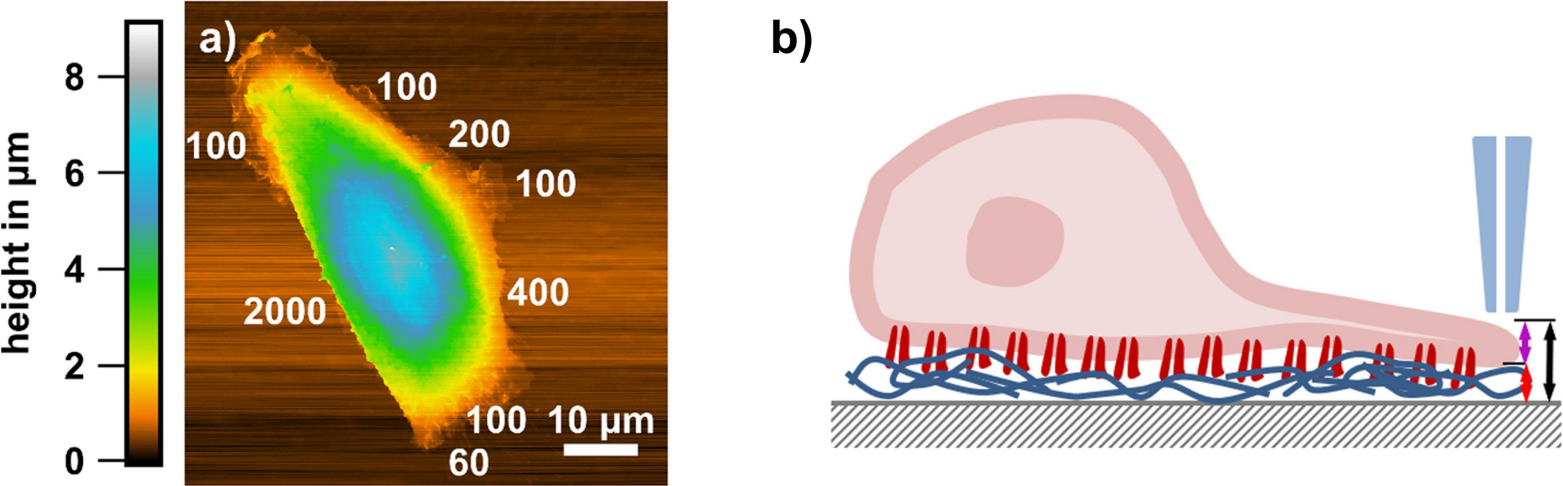
Edge height analysis. (a) Topography of a fixed osteoblast on a 10 nm Au layer on glass with indicated results (in nm) of step edge height analyses in the rim region. Cells show vastly differing edge heights, depending on the macroscopic location. However, the transition from regions of large edge heights to smaller ones is rather smooth. (b) Scheme of the measuring principle of the step edge heights. We determine the overall height of the edges (black arrow), which contains the cell thickness at the rim (purple arrow) as well as the gap between substrate and basal cell membrane (red arrow).

In Figure 9a we show a typical example of the rim step edge height of an osteoblastic cell. Other MG-63 cells exhibit similar edge heights, though the weighting of the different height regimes measured varies with the overall shape of the cell. The step edge heights were systematically measured at the rim of the lamellipodia as well as at ordinary edges of the cells. Note, that the step edge height not only incorporates the gap between cell and surface of the material but also the local height of the cell itself (Figure 9b). Both parameters may vary independently, and from the three-dimensional cell morphology we can only determine the sum of gap and cell height.

At the higher edges (above 1 µm) the step edge slope is greater than 89° and there is hardly any curvature detectable on the cell before its height falls to the substrate level. This could mean that the cell has a pronouncedly undermined configuration, compatible with the gap distance rising at the rim of cells (see discussion below). The flatter cell edges do show slight curvatures on top and at the ledge side, and slope angles measure down to 45°. The smallest heights at homogeneous regions measured about 100 nm (Figure 9a). Taking into account that the ECM takes already about 60 nm [48], the curvature radius of the membrane would correspond to 20 nm. Values in this regime are in line with protein-aided cellular membrane curvatures. For example, intracellular membrane tubes exhibit diameters of a few tens of nanometers, a maximum curvature is assumed to be generated via the Bin/ampiphysin/Rvs (BAR) protein and corresponds to less than 1/(10 nm) [49–50]. Sometimes we find rugged traces with only 60 nm height. We suspect that those may originate from protein secretions from the extracellular matrix left behind, material that was formerly present within the gap between cell and substrate. Either this protein becomes exposed because the lamellipodium has folded away from the surface (to become a peripheral ruffle) or parts of the cell have been retracted from that region in the course of migration. The smallest heights above these minimal heights measure around 100 nm, which we attribute to the height of lamellipodia including the gap. Apart from that, we find relatively high step edges of ca. 1 µm and above, which we attribute to ordinary or trailing edges of osteoblastic cells (Figure 9a).

An elegant method to measure the cell–surface gap at confocal resolution is metal-induced energy transfer (MIET) where the fluorescence lifetime map of a membrane label is transferred into a gap distance map [48]. The cell is adhered to a transparent metal layer and gap distances of 67 ± 7 nm have been determined for fibroblast-like cells (A549). Interestingly, the gap varies by more than 40 nm along the adhesion interface and by more than 70 nm in space and time. At the peripheral region larger gaps have been observed. These values do include only the extracellular matrix because the dye is situated in the plasma membrane. Our step edge heights are compatible with these results and point towards lamellipodia thicknesses in the region of 100 to 200 nm for osteoblastic cells.

An alternative approach to measure the thickness of lamellipodia is to measure the thickness (or better the width) of “upright” peripheral ruffles, that is, former lamellipodia, such as shown in Figure 4a. Note that the aspect ratio of the nanopipette probe is rather high and steep slopes up to almost 90° can be reproduced. Such analyses yield thickness values of 180 nm. This can be seen as an upper limit for the height of a fully formed lamellipodium. Thus, the heights at the rim edges of osteoblastic cells vary between 100 nm and more than 2 µm along their circumference, and they are similar on both substrate types.

### Membrane fluctuations

**Figure 10:**
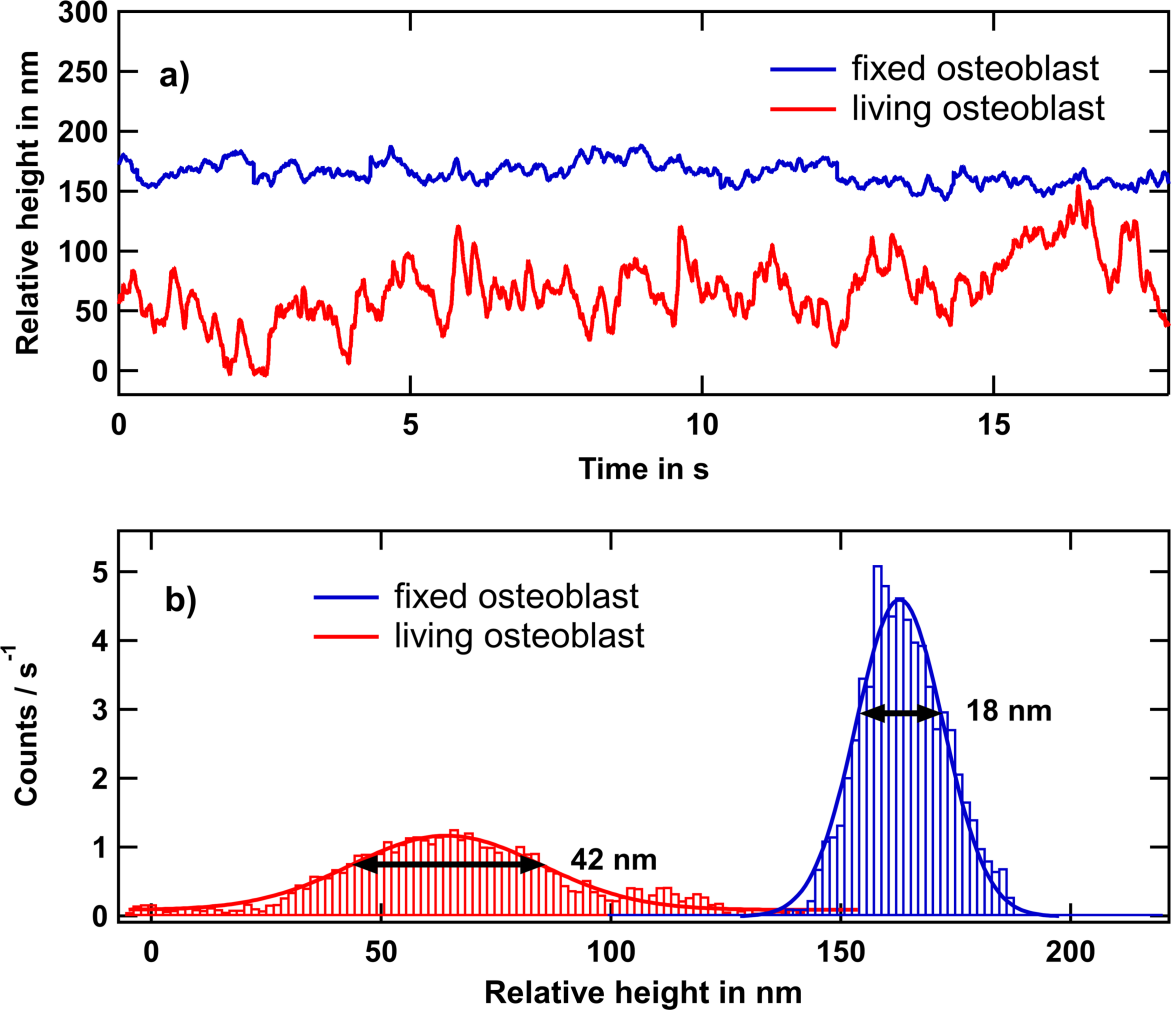
Membrane fluctuations of fixed and living MG-63 osteoblastic cells. (a) Local height variations as a function of the time of membrane displacements of living (red) and fixed (blue) cells. We introduced an artificial height offset to avoid overlapping graphs. (b) Histograms and corresponding Gauss fits of the traces yield standard deviations of 21 and 9 nm for a living and a fixed cell, respectively.

Since topographic measurements were shown not to be sufficient to evaluate dynamic processes such as migration (see Figure 2a,e), we focused on inspecting local apical membrane fluctuations. They might contain valuable information about their origin and give new insights into intracellular dynamics at the leading edge of cells. Thus, they might enable the assessment of, for example, properties of the substrate regarding cellular migration. Especially forward–backward oscillations, so-called “actin waves”, which are important for the formation of lamellipodia, have been shown to dominate membrane fluctuations with characteristic timescales of ca. 10 s and amplitudes of more than hundred nanometers [51–52]. To investigate the apical fluctuations with regard to cellular migration, we selected several positions on the cell (somewhere between the nucleus and the cell edge) and acquired time traces either of current or of height variations. The acquired signal covers a frequency range similar to that of the pre-amplifier bandwidth of up to 1 kHz. For the extraction of root mean square (rms) fluctuation amplitudes the statistical standard deviation of the relative SICM height values (ca. 10^4^ each, Figure 10a) was determined. For better visualization the data was binned and Gaussian fits were applied (Figure 10b).

The resulting standard deviations in case of a living and a fixed cell are 21 nm and 9 nm, respectively. An analysis of a single time traces is shown in Figure 10a. Membrane fluctuation amplitudes turn out to amount to a few tens of nanometers and appear to be substantially larger on living than on fixed cells. Since PFA, via denaturation, only stiffens proteins and leaves nucleic acid, lipids, and aqueous medium intact, one may be tempted to conclude that ATP-driven or metabolic processes are responsible for the non-equilibrium character, that is, the larger extent of live cell membrane fluctuations [53–55]. Especially active membrane motions generated via the spectrin corset underneath the membrane are suppressed when the cell is dead [56]. Membrane fluctuations of fixed cells are assumed to be merely thermally driven. Random samplings showed that live cell fluctuations of osteoblasts exhibit about two times larger rms amplitudes than those of fixed osteoblast cells. This is in line with the results obtained from red blood cells [57], where membrane fluctuation amplitudes were determined via holographic microscopy and found to be seven times larger on living cells than on fixed cells in the frequency range of 0.2 to 12 Hz. A rms amplitude 3.6 times higher on living than on fixed fibroblast-like cells (L929) was reported [39]. However, comparing the apparent fluctuations between living and fixed cells is not straightforward, since not only the bare membrane fluctuations are measured. It needs to be considered that macroscopic cell migration as well as the guided motion of the membrane protrusions itself lead to an increase of the measured fluctuation amplitudes. It has been shown that, for example, microvilli are moved up to 100 nm/s on the surface of A431 epidermal cells [58]. Taking this into account, the high amplitudes with a periodicity of several seconds could partly result from ruffles moving beneath the pipette opening. Slightly higher rms amplitudes may also be due to different temperature because fixed cells are measured at room temperature, while live-cell imaging is carried out at 37 °C.

### Frequency response behavior

**Figure 11:**
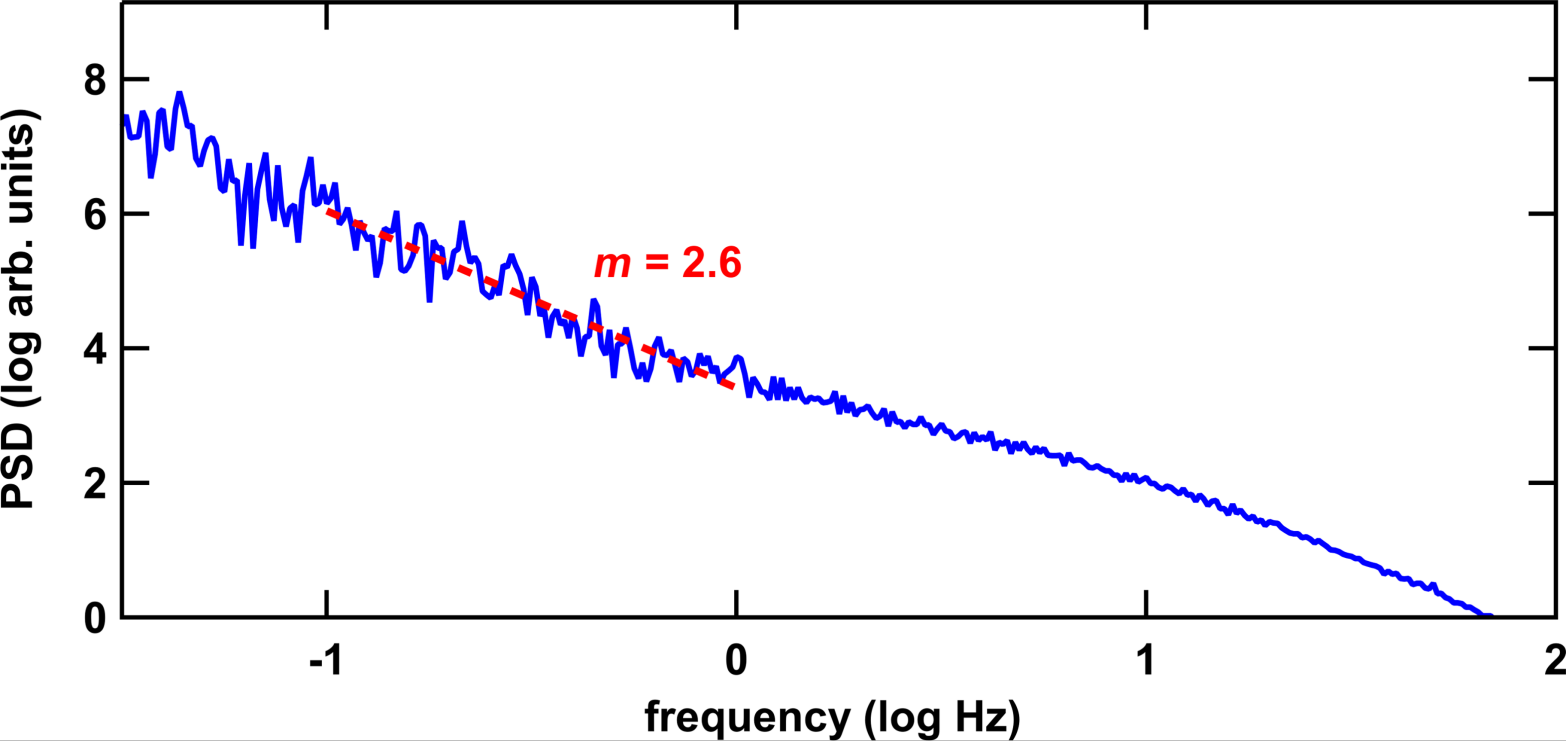
Power spectral density of vertical membrane fluctuations of a living osteoblastic cell. The exponential decay of the spectral density in the region of 0.1 to 1 Hz amounts to *m* = 2.6.

Owing to the fact that morphological changes at the cell surface and cell migration happen rather slowly, they should only contribute to low-frequency fluctuations. To investigate the frequency behavior we focus on measuring the height with activated feedback loop in the frequency range of 0.2 to 500 Hz. Most systems exhibit an *f*^−^*^m^* spectral power density (SPD) with *m* varying between 4/3 and 2, depending on the frequency band [59]. For living eukaryotic cells lower exponents (i.e., higher values of *m*) have been derived [54,60–61]. Indeed, for osteoblasts we observed *m* = 2.48 ± 0.18 in the frequency region of 0.1 to 1 Hz (Figure 11). Above 1 Hz and up to ca. 10 Hz *m* approaches a value around 4/3, which has been associated to the physical effect of hydrodynamic bending of the membrane [60].

As reference we used glass substrates and fixed osteoblasts, which exhibited *m* values of 2 in accordance with viscous stress of the liquid [59]. Subtle deviations from *f*^−^*^m^* can be observed sometimes, that is, there are varying exponents at low frequencies for time periods longer than a couple of seconds. Those may originate from migration. However, extensive statistics that take into account the measurement location on the spreading cell are required. Nevertheless, extracting the fraction of active, that is, ATP-depending fluctuations with spatial and frequency band resolution may help to develop cell activity parameters for the assessment of cellular programs such as adhesion on materials surfaces.

## Conclusion

High-resolution SICM topographies of living and fixed MG-63 osteoblast cells reveal the three-dimensional morphology and dynamics of their cell membranes under largely noninvasive conditions. The most striking feature observed is the large amount of sheet-like plasma membrane protrusions, that is, dorsal and peripheral ruffles. Dorsal ruffles vanish to a large extend at cell rim regions when spreading is promoted and the cells exhibit large adhesion areas (above 2500 µm^2^), that is, on amine-covered substrates, leading to smoother regions towards the lamellipodia. This suggests that ruffles act as membrane storage. However, we cannot discriminate between the scenario in which the cell anticipates rapid adhesion, that is, the ruffled membrane is brought towards the cell rim in order to establish lamellipodia and the scenario in which the ruffles remain behind after unsuccessful lamellipodia formation. Yet, the former scenario is rather unlikely since cells in the early stages of adhesion (low adhesion area) on PPAAm coating did not show a higher ruffle density than those on glass.

Heights at the rim edges of the osteoblastic cells vary between 100 nm and more than 2 µm along the circumference. The smallest heights of around 60 nm may be due to ECM material left behind after the cell has retracted from such positions. Small heights of a few hundreds of nanometers may refer to lamellipodia regions, while the larger heights of a few micrometers and less curvature are typical for an ordinary configuration at cell rims.

Membrane fluctuation analyses reveal larger displacement amplitudes for living than for fixed cells. Looking at the frequency space of membrane fluctuations, living osteoblast show slightly higher absolute scaling exponents of the power spectral density than fixed cells. However, up to now it is not clear whether these characteristics are specific for different locations, for example, cell nucleus or lamellipodia or whether they even differ for various substrates offered to the cells, which would mean these exponents are cell-program specific.

## Experimental

### Substrate preparation and characterization

Either pristine borosilicate glass or coatings of positively charged plasma-polymerized allylamine (PPAAm) or negatively charged Au were used, see below. Viability tests (MTS) were performed on glass, sputter-deposited Au, polydimethylsiloxane (PDMS), and PPAAm coating with native titanium and cell culture plastic as reference. Cell viability turns out to be similar on all of these substrates. The spreading area was assessed after 1 h using PKH membrane dye. This revealed almost twice as much adhesion area for the PPAAm coating (ca. 1500 µm^2^) compared to other substrates (Au, Ti with 820 µm^2^). On PDMS the spreading after 1 h was very poor (only 200 µm^2^). Therefore, we refrained from further utilizing PDMS substrates.

Zeta potential and water contact angle were measured to be 8.6 mV and 68°, respectively, for PPAAm [62]. In case of the Au layer, −119 mV and 101°, respectively, were determined.

Zeta potential measurements were performed using the SurPASS™ system (Anton Paar, Ostfildern, Germany) to determine the surface potential. Au- and PPAAm-modified titanium substrates were placed in pairs in the measuring chamber with a gap height of 100 μm. The streaming potential was measured at pH 6.5 to 8.0, at 150 mbar in a 1 mM KCl solution (VWR International, Darmstadt, Germany). Zeta potential values were determined with the associated software Attract 2.1 (Anton Paar, Ostfildern, Germany) according to the Helmholtz–Smoluchowski equation. Zeta potential values at pH 7.4 were calculated with the function “linear regression” using the software GraphPad Prism Version 6.05 (*n* = 3).

Water contact angle values were obtained by the sessile drop method using the drop shape analyzer DSA25 (Krüss, Hamburg, Germany). Drop shape images of 1 µL water drops were acquired with the digital camera of the DSA25 under atmospheric conditions at room temperature (*n* = 3). Water contact angle values were calculated with the associated software (ADVANCE, V.1.7.2.1, Krüss, Hamburg, Germany) via Young's equation.

Plasma polymer coating: The specimens were coated with a plasma-polymerized allylamine (PPAAm) nanolayer using a low-pressure plasma reactor (V55G, Plasma Finish, Germany) in cooperation with the Leibniz-Institute for Plasma Science and Technology (INP) e.V. Greifswald according to a two-step procedure described earlier [3–4,11]. Briefly, PPAAm was deposited by a microwave-excited (2.45 GHz) pulsed plasma (500 W, 50 Pa, 50 sccm Ar) for 480 s (effective treatment time to initiate plasma polymerization of allylamine).

For Au coating a sputter coater (Cressington 108 auto/SE, UK) was used. The sputter parameters were Ar pressure: 0.06 mbar, sputter current: 30 mA, time: 60 s, and distance between sample and target: 55 mm. The thickness of the coating was measured online by a quartz crystal thickness monitor (Cressington MTM 10, UK) and the sputter process was stopped at a nominal value of approximately 10 nm. The Au layers were only applied to half of the glass area in order to create an in situ reference. Since we observed spread cells adhering directly across the border between Au and glass, the cells apparently exhibited no preference.

### Cell culture

For the SICM experiments, human osteoblast-like cells of the cell line MG-63 (American Type Culture Collection ATCC^®^, CRL1427™, Bethesda, USA) were used. This cell line has been successfully applied for studying cell–material interactions [63] with similar characteristics to those of primary human osteoblasts [64–65]. Cells were cultured in Dulbecco’s modified Eagle’s medium (DMEM, 31966-021, Life Technologies Limited, Paisley, UK), with 10% fetal calf serum (FCS, Biochrom FCS Superior, Merck, Darmstadt, Germany) and 1% antibiotics (gentamicin, Ratiopharm, Ulm, Germany) in a humidified atmosphere at 37 °C with 5% CO_2_. The differently coated borosilicate glass slides (22 mm × 22 mm) were cleaned with ethanol. Cells were seeded with a density of 4000/cm^2^ and cultured for 24 h. This rather low density largely guarantees single cells, which are desirable for investigation of cell edge heights. For comparison and resolution enhancement in SICM analysis the cells of some samples were fixed. For doing so 4% paraformaldehyde (Sigma-Aldrich, St. Louis, MO, USA) was applied for 10 min at room temperature.

### Measurement principle and data preparation

Scanning electron microscopy (SEM) was performed using a field-emission SEM (Gemini Supra 25, Zeiss) at 1 keV electron energy without Au coating.

For SICM a commercial AFM/SICM setup (NX-bio, Park Systems, Korea) with a live-cell chamber (5% CO_2_, 37 °C) was used. The sample was immersed in physiological electrolyte (DMEM with 10% FCS and 1% gentamicin) in case of living topography and membrane fluctuation measurements. Measurements of fixed cells took place at room temperature (21 °C) in phosphate-buffered saline (PBS).

Nanopipettes with opening diameters below 80 nm were pulled from borosilicate capillary tubes (inner diameter 0.58 mm) using a CO_2_ laser puller (Sutter P-2000, USA). The used parameters were Heat: 260, Filament: 4, Velocity: 50, Delay: 225, and Pull: 140. The opening diameter was exemplarily determined by SEM and via the measurement of *I*–*V* characteristics [21]. The bias voltage applied between pipette and bath electrode was approximately 100 mV. Both electrodes were non-polarizable (Ag/AgCl).

For SICM topography measurements the nanopipette approached the surface until the setpoint of 0.98 nA (corresponding to an ion current reduction of 2%) was reached. After that, the pipette was retracted for a couple of micrometers with respect to the latest acquired surface height and moved laterally to the next scanning point. The reference current was measured, followed by re-approaching until the setpoint was reached again. This is referred to as hopping, approach–retract, or dynamic scanning to avoid crashes at steep edges due to the lateral insensitivity of the pipette. The error signal, that is, the difference between setpoint and actual ion current, should be small and was taken as co-image. For membrane dynamics measurements the nanopipette was kept at constant lateral position over the cell and either temporal height variations with activated feedback loop or current variations at deactivated feedback loop were acquired. Temporal current and height spectra were evaluated using the Igor Pro software (WaveMetrics, Inc.).

As main observables we used the nanomorphology and dynamics of lamellipodia and rim features of adhered osteoblasts in the initial phase of cellular adhesion. SICM data evaluation was performed using Gwyddion and Igor Pro. A plane was subtracted from the raw data to cancel out sample tilt and long-term drift. The different rows along the fast scan direction were aligned by adjusting the row offset such that the median value of the difference between two neighboring rows equaled zero. The images shown in Figure 2a and Figure 2e were further processed by subtracting 2D polynomial backgrounds using Igor Pro to emphasize small features. Furthermore, the glass surface shown in Figure 2a as a black area on the right side was artificially set to height “0”. Colors in 3D rendered images are a combination of simulated illumination and height information by using Gwyddion’s 3D view (option overlay checked). Surface areas, *A*_eff_, and projected areas, *A*_proj_, were determined using Gwyddion's built-in statistical analysis feature, which computes pixel-based sums and, thus, yields numerical estimates of *A*_eff_ and *A*_proj_.

Frequency plots were evaluated from raw data using the FFT algorithm with a Blackman window in Igor Pro. Bright-field microscopy was carried out with an inverted microscope (Nikon ECLIPSE Ti-U, Japan) from below through the glass slide. Since the lateral SICM frame sizes are rather small, an optical overview image was taken from below in order to place the nanoprobe at the selected position from top. After that, light was switched off to keep potential field triggers low. A second bright-field image was taken after SICM acquisition, in order to ensure spatial assignment in case of migrating cells.
